# Intimate Partner Violence among Canadian Muslim Women

**DOI:** 10.1177/08862605211021516

**Published:** 2021-07-12

**Authors:** Maryam S. Alghamdi, Bonnie K. Lee, Gabriela A. Nagy

**Affiliations:** 1 Faculty of Health Sciences, University of Lethbridge, Lethbridge, AB, Canada; 2 Department of Psychiatry and Behavioral Sciences, Duke University School of Medicine, Durham, NC, USA; 3 Duke University School of Nursing, Durham, NC, USA

**Keywords:** IPV, Canadian Muslim women, Immigrant, 1st and 2nd generations immigrants, adverse childhood experience

## Abstract

An examination of the interaction of pre- and post-migration stressors is critical to understanding Canadian Muslim immigrant women’s experience of intimate partner violence (IPV). This study uses a dominant qualitative design, supplemented by quantitative data to understand eight Canadian Muslim immigrant women’s experience of IPV from six countries of origin. Five themes were identified: (a) childhood exposure to trauma and violence, (b) iron cage of society, (c) the fusion of love and violence, (d) post-migration challenges and assistance, and (e) toll and consequences of IPV. These themes are described to illustrate the trajectory in the development of IPV and the participants’ eventual decision to leave their relationship. Pre-migration experiences included adverse childhood experiences, family history of IPV, and difficulty with help-seeking for IPV. Post-migration challenges of language difficulties, lack of social connections, internalized familial patriarchal values, and sexism influenced women’s help-seeking and decision-making. Results from this sample suggested that immigrant Muslim women are likely more affected by IPV in comparison to Canadian-born Muslim women, experienced more stressors, less support, delayed help-seeking process, and more serious mental health consequences. Quantitative measures revealed negative effects of IPV on women’s mental and overall health. The roles of ethnic communities, religious institutions, law enforcement, and service providers in supporting Canadian Muslim women with experience of IPV are discussed.

## Introduction

Violence against women, identified as a public health problem and a human right issue, has been affecting more than one-third of women worldwide (WHO, 2020). In Canada, intimate partner violence (IPV) constituted approximately 30% of all police reported violence (Burczycka & Conroy, 2018). IPV affects women disproportionally, as eight in ten women experienced IPV as the most common type of violence (Burczycka & Conroy, 2018). Globally, between one-third to half of all femicides are committed by an intimate partner (WHO, 2020).

One important sub-group of interest is Muslim women residing in Canada who represent a rapidly growing segment of the total Canadian population (National Household Survey, 2011). Based on the most recent national statistics (National Household Survey, 2011), more than 1 million Muslims live in Canada and comprise 3.2% of the national population. Further, Muslims represent the second largest religious group after Christians, consisting of various distinct ethnic groups originating in many countries, principally Asia and Africa (National Household Survey, 2011). Moreover, almost seven in ten (68%) Muslims in Canada are foreign-born (National household Survey, 2011), suggesting a large proportion of the total population is in the process of resettlement, acculturation, and integration.

Unfortunately, there is limited data regarding police-reported incidents that are specific to the experience of IPV among Canadian Muslim immigrant women. Nevertheless, approximately 25% of immigrant women, especially Arab, Iranian, and Afghani women, report a lower sense of safety, compared with Canadian-born women (Burczycka & Conroy, 2018). The lack of data on IPV among Muslim women in Canada is worrisome, considering that Muslim women are one of the largest minorities that is rapidly growing, hence there is an increase in demand for more culturally sensitive practices in Canada to address IPV in this population.

### Acculturative Stress

Acculturative stress refers to a decline in health status including psychological, somatic, and social aspects that can occur in individuals who are undergoing the process of acculturation (Berry et al., 1987; Schwartz et al., 2010). Acculturative stress can arise from loss of social support and connections, language difficulties, financial hardships, and changes in the gender roles and family dynamic. Data from the Survey of Muslims in Canada suggest that approximately a third of Muslims in Canada have experienced discrimination or unjust treatment based on their religious identity (Neuman, 2016). Approximately 36% of police-reported hate crimes were motivated by religion and Jewish and Muslim people were the main targeted groups. The data also suggested that Muslim women, who wear a head cover, experience more religious discrimination than Muslim men (Neuman, 2016).

### Loss of Social Network

The process of immigration can lead to relationship/family disturbances, decreased communication, and isolation at a time of great vulnerability (Tate, 2011). Muslim immigrant women residing in Canada report low levels of social support, stigmatization, and are more likely to tolerate IPV and less likely to report it to the authorities (Baobaid, 2002).

### Financial Strain

Research indicates that under-employment is a risk factor for IPV^1^ (Eamon & Wu, 2011; Kimerling et al., 2009). Specifically, the disadvantages that immigrant women face with under-employment and under-paying jobs may compound other life stressors that increase the risk of IPV (Barrett & Pierre, 2011). When tension and conflict arise, immigrant women have limited financial resources to cope with those stressors (Barrett & Pierre, 2011). Additionally, experiencing IPV can incur financial costs to survivors as it can lead to loss of productivity and absenteeism^2^ (Aizer, 2010) and presenteeism^3^ (Maskin et al., 2019).

### Patriarchy and Sexism

Patriarchal values, that is, institutionalized gender inequalities that legitimize male domination and female submission (Hunnicutt, 2009) and sexist norms (i.e., negative attitudes toward women; Pease, 2019), are rampant globally (Johnson, 2005) but may be especially relevant to Muslim women migrating to Canada. Specifically, a number of studies suggest that increases in individual autonomy and freedom (e.g., employment status, lower restrictions on mobility, fewer cultural and religious obligations), loss of ethnic and gender identity, and changes in gender roles and responsibilities in the household result in shifts of gender power dynamics and familial relationships in the post-migration context thereby affecting family dynamics (Hyman et al., 2011; Kasturirangan et al., 2004; Mahapatra & DiNitto, 2013; Raj & Silverman, 2002). Those changes might be perceived by a male partner as threatening to his status as the head of the family, especially if combined with loss of employment, under-employment, experience of racism and discrimination (Baobaid, 2002; Milani et al., 2018).

## Study Aims

The purpose of the present study was to understand the experiences of Canadian Muslim immigrant women surviving IPV by analyzing data obtained through semi-structured interviews of eight participants who were ethno-culturally diverse. The aims were two-fold: (a) to understand the effects of familial, cultural, religious, and societal influences on IPV and (b) to describe the trajectory and experiences of women survivors of IPV in a post-migration context influenced by their pre-migration experience.

## Theoretical Framework

Straus’ multi-factorial General Systems Theory for family violence guided the conceptualization and development of the interview questions for this study (Gelles & Straus, 1979; Straus, 1973; Straus & Hotaling, 1980). The theory views family violence as the product of a positive feedback social system operating at the individual, family, and societal levels. The theory assumes that isolated cause-effect analyses cannot capture the complexity of social behavior such as family violence but a theory must consider the complexity of mutually influencing factors. Straus and Hotaling (1980) identified seven interacting factors at the individual, family, and society levels that condone violence and these values and beliefs become a script internalized into personality and behavior.

## Methods

### Sampling and Recruitment

Institutional ethics approval for the procedures and protocols in conducting this study was obtained before its implementation. Canadian Muslim immigrant women who experienced IPV were purposively selected using convenience and snowball sampling techniques. To gain access

to potential participants, the first author contacted various immigrant services, Muslim community centers, mosques, and end violence against women organizations in a major metropolis in Canada. The organizations’ role was to assist in identifying potential participants and sending out an invitation letter and putting up posters announcing the study in their newsletter or website to explain the purpose of this study.

### Eligibility Criteria

The inclusion criteria used in the recruitment for this study were: Canadian Muslim women ages 18 to 60 who had experienced control, mistreatment, or harm by a romantic partner. Participants had to speak and read English or Arabic and have the ability and willingness to give informed consent. There were no exclusion criteria.

## Measures

### Quantitative Self-Report Measures

**Demographic Questionnaire.** A demographic questionnaire inquired about age, religion, and frequency of practicing religion, education, employment, partner employment status, marital status, and years of marriage, country of origin, length of residence in Canada (in years), household income, and their partner’s income.

Hurt, Insulted, Threatened with harm, Screamed (HITS) Scale. HITS (Sherin et al., 1998) is an IPV verbal and physical abuse screening tool comprising four items. Items are rated on a Likert-type scale ranging from 1 (never) to 5 (frequently), with a total range of 4–20. Any score greater than 10 is considered positive indication for IPV. This measure has indicated good internal consistency (Cronbach’s alpha = 0.80) and concurrent validity with a measure of verbal and physical aggression (correlation = 0.85; Sherin et al., 1998).

Generalized Anxiety Disorder Scale (GAD-7). The GAD-7 (Spitzer et al., 2006) measures anxiety symptoms (e.g., nervousness, worry, trouble relaxing, and annoyance) occurring in the prior 2-week span. This measure has demonstrated very good test-retest reliability (intraclass correlation = 0.83)., internal consistency (Cronbach’s alpha = 0.92), and good procedural validity or agreement between self-report and clinician (intraclass correlation = 0.83; Spitzer et al., 2006).

Patient Health Questionnaire (PHQ-9). The PHQ-9 (Kroenke et al., 2001) inquires about experiences with depression symptoms (e.g., mood disruption, anhedonia, appetite and sleep changes, self-esteem, and loss of energy). This measure has indicated excellent internal consistency (Cronbach’s alpha = 0.86–0.89) and has also demonstrated adequate criterion and construct validity when using other similar outcomes (Kroenke et al., 2001).

Primary Care PTSD Screen (PC-PTSD). The PC-PTSD (Cameron & Gusman, 2003) is a four-item screening tool that examines four symptoms of post-traumatic stress disorder (PTSD) including re-experiencing, numbing, avoidance, and hyper-arousal. This screening tool has demonstrated adequate sensitivity (0.76–0.85) and specificity (0.71–0.88) (Bliese et al., 2008).

Short-Form General Health Survey. A single item “In general, how would you describe your health” from the Short-Form General Health Survey (SF-36; McHorney et al., 1993) was utilized to tap into the construct of self-reported health. Responses are rated on from 1 (excellent) to 5 (poor). A validity study found that the entire SF-36 has demonstrated satisfactory internal consistency (Cronbach’s alpha > 0.7; Anderson et al., 1996).

### Qualitative Semi-Structured Interviews

In-depth semi-structured interviews were conducted in 2017, majority in English, but also Arabic, depending on the participants’ preferences. Each interview lasted an average of one to two hours by the first author who conducted and transcribed the interviews. The interview questions were generated from the theoretical framework and the literature review. It consisted of broad open-ended questions to give the participants the opportunity to have their voices in the research process. The questions were pilot tested with my supervisor and colleagues to assess the clarity and relevance of the questions and to establish validity. Further, the researcher insured that the location of the interview is comfortable and safe for both the interviewer and the participant. The questions were focused on aspects of the women’s experience, including immigration, intimate relationship, marital conflicts, conflict resolution process, community engagement, childhood, social and religious values, and help-seeking process.

## Data Analysis

### Qualitative Thematic Analysis

The goal of qualitative research is to provide a rich understanding of the phenomenon under study rather than aim for generalizability (Neuman, 2011). A sample size of eight participants was deemed adequate for this exploratory study, as it falls within the range of previous IPV studies with similar aims (Senter & Caldwell, 2002) and similar data were being generated with *N* = 8 suggesting saturation. Themes were identified and described based on salient aspects of the data (Rubin & Rubin, 2011; Sandelowski, 2000), and refined with help from the second author.

## Results

### Study Participant Demographic Information

Individual participant demographic characteristics using pseudonyms are presented in Table 1. All participants identified as female and most (seven of eight participants) were born outside of Canada. The average age was 43 (range of 29–57 years). Excluding the one participant who was born in Canada, the average length of residence in Canada was approximately 12.5 years, with a range of 5–27 years. The average length of IPV relationships was 13 years (range of from10 months to 26 years).

**Table 1.** Participants Demographics.

Name	Age	Country of Origin	Length of Residence in Canada (Years)	Marital Status	Children	Length of Abusive Relationship	Education (Degree)	Employment
Sallam	47	Morocco	6	Divorced	0	6 Years	Bachelor’s	General labor
Khadija	57	Iraq	27	Divorced	0	21 Years	Bachelor’s	General labor
Fatima	47	Iran	5	Separated	2	21 Years	Bachelor’s	Health care
Sherine	38	Canada	38^a^	Single	1	10 Months	Master’s	Health care
Salma	30	Afghanistan	8	Separated	0	5 Years	Bachelor’s	Not employed
Zahra	29	Iraq- Kurdistan	7	Separated	2	9 Years	Bachelor’s	Not employed
Safia	39	Iran- Kurdistan	22	Divorced	1	13 Years	High School	Not employed
Noor	54	Iran	13	Divorced	1	26 Years	Bachelor’s	Educational services

*Note*. ^a^ Participant was born in Canada.

### General and Mental Health Consequences

Participants demonstrated elevated IPV rates on the HITS, with a mean score of 16.1 beyond the cut-off score of 10 indicative of IPV. Notably, the Canadian-born participant reported the lowest score on the HITS. Study participants generally reported severe levels of anxiety, with the mean score on the GAD-7 of 12.6. Study participants demonstrated moderately severe depression symptoms, with a mean score of 14.6 and a high likelihood of experiencing PTSD symptoms, with a mean score of 2.9 (out of a total of 4). Overall, participants reported good general health, with a median score of 3.5 (out of a total of 5). All participants had terminated the relationship either temporarily or permanently at the time of these questionnaires which could have affected their answers. Nonetheless, these quantitative results corroborate with the qualitative findings that the women endured a significant level of abuse with a toll on their mental health with exhibition of severe symptoms of anxiety, depression, and a probable risk of developing PTSD. The quantitative results etch out more sharply the profiles of these women and the degree of their suffering as described in the qualitative section. Combining the qualitative and quantitative data add to the trustworthiness of the overall findings of this study through triangulation.

### Trajectory of Development and Extrication from IPV

#### Theme 1: Childhood of Trauma and Violence.

**Women’s adverse childhood experience.** In this study, all of the participants reported some form of childhood trauma that included: six lived in a war zone, four were exposed to domestic violence, and two experienced loss of a parent during childhood. In addition, father’s multiple wives, father’s excessive alcohol consumption, teen migration were all childhood and pre-migration trauma that the participants expressed. An example was provided by Sallam:

What to tell you, I was raised with my father and my stepmother, my mother died when she gave birth to me, my stepmother had a very hard time with my dad, he married another woman and he used to hit her a lot, and she (thank God) had a job. You know, problems, another life, and no rights.

Fatima grew up in a household where her father verbally abused her mother, and her mother responded in silence and acceptance, therefore she fears her husband, modelled by her mother:

I remember when I was a child it was very bad. I think because, maybe I learned to fear my husband, always thought if I was a little more brave, I can stand in this life. My mother didn’t do anything against the violence, then maybe I learned from her. I remember my father always put down my mother.

Similarly, being physically abused by her father as a child, Sherine accepted physical abuse from her intimate partner:

I maintain a relationship with my father and obviously, he did physically abuse me, so, for me to be able to accept that from the first and foremost important man in my life, it becomes a little bit easier to accept it from a romantic partner.

**Partners’ adverse childhood experience.** All of the participants provided some details of their partners’ childhood trauma that included loss and abandonment, neglect, physical and verbal abuse, living in a war zone, teen migration, poverty, illiteracy, and child labor and imprisonment. Sallam described her partner’s traumatic childhood experiences:

I know that my husband has a complex. Since he was a child, he did not have a mother or father. He grew up with relatives. He immigrated when he was 17 years old. He went everywhere—to Libya, Kuwait, Russia, then Canada. He had to marry an older lady to do his papers. I mean, he grew up in a complex society, they have a lot of problem and now the war, in addition to the internal war.

Zahra reported her husbands’ experience of physical abuse in childhood. “Yes, his father and mother beat him a lot, but he doesn’t like to talk about it. I mean, his mother once burned his hand, there is a still a scar of that burning.” Salma highlighted that poverty and illiteracy were important factors to be considered when looking at her husband’s childhood:

As a kid, he lost his dad, he is the youngest among 12 other siblings, and they were very poor and uneducated, and he was mentally and physically abused by his brothers… his oldest brother told him, “If you got married, you have to beat your wife from the first night, so she will obey you after that.”

Partner’s experience of high level of childhood trauma was an important factor in repeating the abusive behavior in marriage, as Safia reported, “One day when we were together arguing, he pressed my hand, and I said you are breaking my hand, and he said ‘So? My dad broke my mom’s hand, and you are not better than my mom’.”

In summary, all of the women and their partners suffered adverse childhood experiences ranging from living in violent war zone countries to witnessing or experiencing familial verbal and physical abuse, physical and emotional neglect, loss of parents or abandonment, to extreme poverty.

#### Theme 2: Iron Cage of Society, Religion and Culture.

Except for Sherine, the Canadian-born participant, all participants came from collectivist societies and cultures, where particular social structures and networks both positively and negatively influenced the families’ lives. Canadian immigrant women came from traditional Muslim-majority countries where the religion and culture are mutually reinforcing. On the positive side, participants cited respect of the elderly, a tight supportive social network, and family gathering and helping others. As Safia said, “Back home is really tight community, everyone knows everyone, friends, and family; if something happened, they help each other.” These social networks also functioned to enforce cultural values, beliefs, and expectations of the men and women even post-migration.

**Negative social regard for women and divorce.** With the exception of the Canadian-born participant, Canadian Muslim immigrant women had experienced low social regard in their country of origin and in Canada, and even more so if they were divorced. Canadian Muslim immigrant women reported that they had to choose between staying in an abusive relationship, or fleeing violence and being ostracized by their family and community. Khadija stated,

My family do not like divorce, everyone said, ‘Be patient, be patient,’ and when I got married my family were very happy for me, so how would I get divorced in the first month.… At least now they will say, I was patient for 20 years.

Khadija internalized the cultural expectations of her family and lived by the same rules even after immigration. A mother-in-law advised her son to hit his wife to prove his manhood as related in Selma’s words, “His mother once told me after he had hit me so hard: ‘His father did the same thing to me, you are a woman, and you should keep quiet’.” Five out of eight participants reported that the partner’s family maintained the patriarchal extended family system.

**Different gender roles and responsibilities.** Gender inequality was highlighted by all the participants, even the Canadian-born participant, as a contributing factor to IPV. Women indicated that they were socialized from their childhood to take on certain roles within the marital institution. As Fatima reported:

I mean they have kind of patriarchy, yeah, the girl should be so, and the boy should be, you know, we have so many should and should not. Roles about the differences between men and women, I remember, I started to write poems when I was in the university, but my father said it was not good for girls.

Even Sherine, the Canadian-born participant, was subjected to the gender roles that were enforced by her immigrant parents:

Here was the stereotypical patriarchal positions and even for myself, you know, I remember wondering, you know, why? I have a brother who was 18 months younger than me, and I wondered why he was allowed to go outside to play? And my parents expected me to stay inside and clean the table and the dishes, so there were a lot of that going on as well, my father and mother both believed in physical punishment.

Further, the patriarchal system in the household is strongly implemented under the cultural values and reinforced through members of the society. The participants’ partners were shamed if they helped in the housework. For example, Salma stated “Me and my sister-in-law do the housework, my husband (laugh), no way, his mom would shame him if he tried to help with the house work.”

**Household work division and decision-making post-migration.** During the time of the relationship, all of the participants were employed outside the home, worked an equal number of hours as their partners, and contributed to the household income. However, only the Canadian-born participant expressed an equal division of the household work, while the Canadian Muslim immigrant women stated that their partners made all decisions regarding different aspects of their lives. Additionally, they adhered to the previously learned divisions of roles and responsibilities, and the patriarchal division of household work was maintained in Canada. For example, Sallam said, “I go back from work, and he makes me clean the table and wash the dishes, and such, and he curses me in front of his children.”

#### Theme 3: Fusion of Love and Violence.

Patriarchal values and structural gender inequalities in addition to childhood trauma and abuse shaped the early life experiences of the participants and their partners. Having a romantic partner creates an attachment that provides comfort for both the man and the woman.

The male partners were deft in using different tactics of pursuit and courtship to increase emotional and physical closeness to their women before marriage. The women expressed the intensity of their attraction and their strong attachment needs during the honeymoon phase of their relationship. The euphoric state quickly deteriorated after marriage when their male partners started distancing themselves. Sherine expressed, “He would stay out late or exclude me, or just I wouldn’t hear from him for several days, the most was maybe 5 days.” Similarly, Zahra shared, “He is always very distant, I actually don’t feel like I have a husband, we lived in the same house, but he was miles away in his head.”

This increase of emotional detachment developed quickly after marriage and created a mixture of love and violence that coexisted in the relationship which was confusing for the participants. Their attachment needs acted as a powerful motivator for overlooking the warning signs for IPV and remaining in the relationship. For instance, Noor expressed, “I knew what I was getting into but because we had become so close, and I have become so close physically that’s why I was not able to give up, and I was attached emotionally.”

Further, participants’ level of commitment and their hope for change were important factors in their decision to stay or terminate the relationship. Noor stated, “I thought I was in love and I couldn’t stop. I knew what I was getting into, but I said he might change, I may be able to change him.” This suggests that the more the woman is committed to her relationship, the more tolerant she will be of abuse, and hence the more reluctant she will be to leave her partner.

The women expressed an overwhelming sense of regret over their emotional, physical, and financial investments in the relationship. Sallam said, “I didn’t cry over him, we are divorced, but I cried over myself, how I knew someone like this? Are there people like this?” Similarly, Khadija related, “He used me, he used my kind heart, and I loved him so much [crying]. Oh almighty God! I have never refused any request from him.” The sense of regret was further extended to the other areas in their lives like their physical and psychological health, education, financial situation, and social life. For instance, Noor expressed how her physical health improved dramatically after terminating the relationship, and how she felt remorseful that she did not end it earlier. Sherine and Sallam both realized the cost of staying in the relationship on their career, “It was a negative influence, just because, it took up so much of my energy, I actually feel like, the whole duration of the relationship was like a setback for me in terms of my career, my education, my interpersonal relationships, like everything.”

Six of the eight participants entered the relationship with a hope of finding love and emotional stability lacking in their childhood, sacrificed themselves emotionally, physically, and financially to maintain the relationship. Noor, who lost her father when she was 9 years old kept looking for a father figure in a romantic partner. In her own words:

It affected me because I think I was always looking for a father, not a husband. My husband was nearly 12 years older than me when we married, so I think, I didn’t become attracted to boys my age. Why? Because I didn’t have a father, I have always wanted a father, and he knew, and I told him, “You are my father, you are not my husband.”

The mixture of love and violence in the relationship confused them, which led them to keep the hope for reconciliation and change. However, when they terminated the relationship, they experienced a sense of regret for the love they gave out, their unmet expectations, and the lost opportunities in their lives.

#### Theme 4: Post-migration Challenges and Assistance.

Seven participants were immigrants and six had fled a war zone to start a new life in a safe country with freedom and peace. In some cases, the IPV began in their country of origin. Immigration to Canada had a double effect on the women, as the availability of formal resources had positively affected the help-seeking process, but negative experience with IPV services in their countries of origin, underemployment, lack of finances, social isolation and lack of familial support, and internalized cultural beliefs contributed to their tolerance of the IPV and its escalation.

**Pre-migration experiences led to delays in accessing formal resources.** Muslim immigrant women did not seek formal help in their country of origin due to socio-cultural reasons such as service providers’ negative attitude, lack of information and resources, and the lack of encouragement from friends and family to do so. The women reported that these pre-migration negative experiences delayed their help-seeking process in the post-migration context.

**Limiting language skills was a tool of control.** There was a clear distinction between the Canadian-born participant who reached different channels of formal support faster and more easily compared to the Canadian immigrant participants. Some partners restricted their wives’ ability to improve their language level, and thus socially isolated them and hindered their leaving the abusive relationship.

**Abusive partner controlled women through social isolation.** Participants’ partners were reported to use social isolation as a form of control to further isolate them by planting doubts in the women’s ability to live in the Western society on their own, or limiting their contact with family members, or eliminating their contact with Western friends (Raj & Silverman, 2002). The existing literature coincides with these findings as it suggests that abusive partners may use social isolation as a way to further undermine the women’s ability to escape or report the abuse (Ayyub, 2000; Raj & Silverman, 2002).

**Under-employment and low socioeconomic status as.** De-skilling and under-employment were found to increase the risk and intensity of IPV in the post-migration context as the immigrant Muslim women had to accept jobs that did not match their education and had affected them physically, psychologically, and financially. Further, it played an important part in increasing the abuse, due to the financial instability which added more life stress to the relationship and increased tension between the couple. Only the Canadian-born participant had a job that matched her education. Seven of eight of the participants had at least a bachelor’s degree, one was a physician in her country of origin and was de-skilled to a caregiver in Canada. The immigrant Muslim women were financially dependent or manipulated by their partners who controlled their assets and income.

**Formal support and resources.** Most participants indicated that having formal services support helped them in managing the psychological distress and the detrimental effect of IPV. Such support was a determining factor when reporting abuse by an intimate partner. Five participants received police intervention and shelter, six participants received counselling and help from women organizations, and four received welfare, housing, and help with court processing for divorce. Formal support was proven to be a valuable asset in determining the women’s ability to flee the abusive relationship. Sherine expressed how the police and women’s organization helped her:

When I was physically assaulted, I had to contact the authorities, so, once the police came, they decided to press charges and then they advised me on different shelters and mm, I also through this criminal case I was able to receive support through ‘the crime victim assistant program’, and through other organizations such as WAVAW.^4^

Khadija highlighted the important role that the women’s organization played in her life, “The government helped me, and this organization, if it were not for them, I may have ended up homeless.”

#### Theme 5: Toll & Consequences of IPV.

The turning points that led the women to leave were rarely isolated episodes. Even with cases of physical abuse, all the participants had endured an excessive amount of violence and abuse in the relationship to finally decide to leave. Five participants had terminated their relationship for reasons such as continuous physical abuse, extreme physical abuse, infidelity, their children’s wellbeing, and excessive alcohol consumption. Different reasons were provided for staying in the abusive relationship, of which familial and social pressure affected the women’s decision the most. For example, Fatima expressed,

When we wanted to come to Canada, supposed to be, I will stay here, with my children without my husband and I thought maybe it is kind of respectful divorce for me, because it was very hard for me to get divorce in my country.

The immigrant Muslim women were all married and stayed in the relationship for an average of 14 years (range: 5–26 years). Their length of stay in Canada ranged between 5 and 27 years. The Canadian-born participant had a common-law relationship that lasted for 10 months. Canadian Muslim immigrant women cited familial and cultural concerns for staying in the marital relationship whereas the Canadian-born stated emotional attachment as a reason for staying. These findings dovetail with the existing literature that suggests that women are less likely to leave an abusive relationship if there is a lack of social support (Barrett & Pierre, 2011), which applies to the case of the immigrant Muslim women.

**Experience of family pressure and interference.** Our findings suggest that family pressure and interference increased both frequency of incidents and the level of tolerance toward abuse among immigrant women. The Canadian-born participant did not indicate any familial pressure or interference in her relationship. Lack of family support and exposure to violence in the family of origin limited immigrant women’s willingness to discuss the abuse with a family member which added to the stress. The immigrant Muslim women reported receiving little empathy and informal support from their own or their husband’s family and whose input tended to make matters worse. Moreover, participants who expressed a negative family interference, especially from the mother-in-law or older brother-in-law, were at greater risk of IPV due to the pressure on the husband to discipline the wife to please his family. This is in sharp contrast to the Canadian-born participant who did not report any interference from her family in her decisions.

**Differences in experiences of patriarchy and sexism.** Previous research suggests that patriarchy can manifest itself differently in many cultures (Sokoloff & Dupont, 2005). In this study, Canadian Muslim women came from different countries such as Morocco, Iraq, Iran, and Afghanistan (see Table 1). Nevertheless, the patriarchal practices of male dominance and female submission were described by study participants. For example, Canadian Muslim immigrant women expressed the role of the negative social perception of divorced women in hindering their ability to report and to flee the abusive relationship. In contrast, the Canadian-born participant did not express any exposure to negative social perception when she terminated her relationship.

Despite living in Canada, the immigrant women referenced the unfavorable social perception of divorced women in their country of origin, which shows a high level of internalized oppression.^5^ The study findings coincide with previous research that have highlighted the negative social and emotional effects of the social perception of divorced woman (Al-Krenawi & Graham, 1998).

## Discussion

This study added to the limited literature on IPV among Canadian immigrant Muslim women in the post-migration context and its trajectory of development. It included an examination of post-migration stressors contributing to the intensity of IPV among Muslim women and how it affected their help-seeking process. Post-migration stressors such as language training, financial and employment status, extended family reaction to their IPV, internalized sexism and patriarchy, and beliefs about divorce should be included in the history-taking in the assessment of their IPV. Educational programs for abused women and their family members need to be delivered in their own languages and take into account their pre-existing cultural worldviews and adverse experiences. Social perception of divorced women that exists among the women’s ethnic communities and in religious organizations could deter women from breaking away from an abusive marriage. On the other hand, bridges can be built with Muslim ethnic and religious groups to develop them as allies in understanding and preventing IPV among Muslim women and responding to their call for help. This will reduce the likelihood that divorced women with IPV history are marginalized from their own ethnic communities.

The importance of formal resources that are culturally supportive and appropriate when working with IPV cannot be over-emphasized in light of immigrant Muslim women common social isolation and lack of extended familial support. They have also suffered from negative pre-migration negative experience of shame and stigma in reaching out for assistance. Formal sensitivity training for police officers responding to domestic dispute calls is highly needed to understand the cultural differences within the Canadian Muslim immigrant population. Most findings in this study are consistent with the existing literature on IPV, which increased its validity and reliability.

### Support and Expansion of Straus General Systems Theory of Family Violence

The purpose of the present study was to understand the experiences of Muslim Canadian immigrant women who were survivors of IPV. The semi-structured interview guide was informed by Straus’ General Systems Theory (1980), a multi-dimensional theory that views IPV as a product of socialization into violence at the cultural, societal, and familial levels (Straus & Hotaling, 1980). Findings from this study support and expand upon Straus’s theory in a number of ways. According to Straus and Hotaling (1980), male-dominated cultural and religious values contribute to family violence. The women reported witnessing paternal IPV in their family of origin which conveyed the implicit acceptance of IPV as part of normal family life. Straus observed the internalization of these societal and familial values including the fusion of love and violence and the moral rightness of violence for good ends, as in the use of physical punishment of children. These values were incorporated into the personality and behavior scripts of the Muslim women in this study and informed their decision-making process of whether to stay in an abusive relationship or to seek outside assistance. Supporting Straus’s contention that family violence mirrors a larger societal context of violence are the women’s reports of their partners’ early life history in countries of war, dislocation, poverty, complex polygamous families, child labor, and political persecution. Other forms of childhood adversities such as loss and abandonment by parent(s), neglect, poverty, and illiteracy informed the early lives of both the immigrant Muslim women as well as their partners, factors not mentioned by Straus who focused primarily on the perpetration of physical violence. There now exists a growing literature on the role of adverse childhood experiences in the development of adult physical and mental problems as well as experience of IPV (Li et al., 2020; McMahon et al., 2015). The co-occurrence of other forms of adverse childhood experiences with witnessing IPV as children should be noted (Dube et al., 2002). The difficulty of women in extricating themselves from IPV could also be a factor of their unmet psychological attachment needs as a result of adverse childhood experiences as found in this study.

Straus’s General Systems Theory (1980) suggests that lack of formal and informal support, children, and limited financial resources are strong determinants when it comes to leaving or staying in an abusive relationship. A recent study indicates that Canadian immigrant women are more likely to be emotionally abused than Canadian-born women and that first-generation immigrants have a higher level of tolerance toward abuse and less tendency to report (Ahmad et al., 2017). This could likely issue from a combination of factors, including internalized models of patriarchy and violence as well as post-migration stressors identified in this study. These findings go beyond Straus’s theory that did not consider the stress posed by immigration as a contributing factor to women’s vulnerability to IPV. Further our study sheds light on the interaction of old and new scripts of acceptability of IPV between one’s original and adopted culture as a factor empowering of IPV survivors. Our findings highlighted the differences between first and second-generation Muslim immigrants’ readiness to seek help and their degree of tolerance of an abusive relationship with different degrees of mental health consequences. Changing ecological conditions and exposure to other cultural norms and values through immigration raised the women’s awareness of what constitutes IPV, increased their feeling of safety and ability to report, seek help and utilize counselling and housing resources.

## Study Limitations

Our findings are presented in light of several important limitations. First, generalizability of the research findings is limited due to the small sample size in one metropolitan location. Differences may exist between urban and rural areas where stigma, immigrant services, and education may differ (Shannon et al., 2006). Second, we collected some information from female study participants about their male partners whose childhood histories were replete with trauma and adversities. Such data suggest that to better understand the dynamic of IPV within an intimate relationship, both parties’ backgrounds, and viewpoints should be examined. Third, Muslims in Canada are not a homogeneous group and findings from this study reflect the data collected from a sample of Canadian Muslim immigrant women who experienced IPV, and do not necessarily reflect the dynamics of all Muslim marriages.

Future studies are recommended to expand on the differences in the experience of IPV in the pre- and post-migration context with a larger sample that allows for further comparison of first- and second-generation Muslim immigrants. Further, all women in this study were accessed through the help of women’s organizations. However, we have limited information regarding how the women accessed these services, and what raised their level of awareness of such services. Future research is recommended to link available IPV resources with immigrant women in need of such services and how they can access them.

## Authors’ Note

This article is based on Maryam Alghamdi’s Master’s thesis supervised by Dr. Bonnie Lee.

## Notes

1. Under-employment refers to working for minimum wage or working part-time involuntarily.

2. Absenteeism refers to being physically absent from work.

3. Presenteeism refers to feeling unwell while being physically present at work, which may lead to loss of quality and quantity of work.

4. WAVAW stands for Women Against Violence Against Women.

5. Tappan (2006) defined internalized oppression as “a concept used to describe members of marginalized or minority groups who are powerless and often victimized, both intentionally and unintentionally by members of dominant groups, and those who adopted the dominant group ideology and accept their subordinate status as deserved, natural and inevitable” (p. 2116).

## Author Biographies

**Maryam S. Alghamdi** received her MSc in Health Sciences from the University of Lethbridge, Alberta. Prior to her studies in Canada, she worked as a Psychologist in a transition house in Saudi Arabia, where she developed her research interest in intimate partner violence and how it disproportionately affects women who are dealing with additional barriers like immigration, mental health challenges, substance use and homelessness. Currently, she works in the field of mental health support services and as an anti-violence against women advocate.

**Bonnie K. Lee,** PhD, is an associate professor in Health Sciences, Addictions Counselling at the University of Lethbridge and a Clinical Fellow of the American Association for Marriage and Family Therapy. Her research focuses on the impact of adverse childhood experience on couple relationship, addiction and mental health with two completed randomized trials on a systemic intervention Congruence Couple Therapy for these issues. She has also researched processes that aided the successful integration of immigrant women.

**Gabriela A. Nagy,** PhD, is an assistant professor in the Department of Psychiatry and Behavioral Sciences at Duke University School of Medicine and a Clinical Assistant Professor in the Duke University School of Nursing. Dr. Nagy is a health disparities researcher whose recent work has focused on understanding risk and resilience factors that lead to health decays in immigrants as well as interventions that can reduce acculturative stress in immigrants. Her research has been funded by the National Institutes of Health, internal grants, and community foundation grants.
